# Recycling Apparent Waste Into Biologicals: The Case of Umbilical Cord Blood in Italy and Spain

**DOI:** 10.3389/fcell.2021.812038

**Published:** 2022-01-04

**Authors:** Paolo Rebulla, Sergio Querol, Simonetta Pupella, Daniele Prati, Joaquin Delgadillo, Vincenzo De Angelis

**Affiliations:** ^1^ Department of Transfusion Medicine and Hematology, Foundation IRCCS Ca’ Granda Ospedale Maggiore Policlinico, Milan, Italy; ^2^ Banc de Sang I Teixits, Barcelona, Spain; ^3^ Centro Nazionale Sangue, Rome, Italy

**Keywords:** umbilical cord blood, platelets, platelet gel, neonatal transfusion, eye drops, biologics

## Abstract

Most public cord blood banking programs are currently facing financial difficulties due to a progressive decline in the number of cord blood transplants performed worldwide and to a high discard rate of the donated units caused by progressively increasing thresholds of the stem cell dose required to perform safe and effective hemopoietic cord blood transplants. Recycling a proportion of unused cord blood units to prepare novel cord blood components obtained with minimal manipulation (platelets, plasma, red blood cells) and to develop more technologically complex products regulated in the US as Cellular and Gene Therapy Products and in Europe as Advanced Therapy Medicinal Products [e.g. virus-specific T cells (VST), natural killer (NK) cells, induced pluripotent stem cells (iPSCs) is a promising strategy to increase the therapeutic value and reduce the financial deficits of public cord blood banking. Based on encouraging preliminary evidences reported in the literature, additional laboratory studies, large multicenter clinical trials and international regulatory harmonization are necessary to achieve these important goals. This article describes organizational, methodological and regulatory advancements developed in Italy and Spain to promote the clinical use of cord blood platelets, plasma and red blood cells.

## Introduction

During the last 3 decades, newborn’s blood remaining in the placenta after term deliveries has been collected from the umbilical cord, processed and cryopreserved in public cord blood (CB) banking programs implemented following the first successful hemopoietic CB transplants pioneered by Eliane Gluckman, Hal Broxmeyer and Joanne Kurtzberg in the late 1980s. So far, a global cryopreserved CB inventory of about 800,000 units has allowed the performance of more than 40,000 transplants in pediatric and adult patients affected by blood cancers, immune deficiencies, metabolic disorders and hemoglobinopathies (Kurtzberg, 2017; Mayani et al., 2020; Jöris et al., 2021).

In the early years of CB banking, variable proportions of the donated CB units were processed and cryopreserved, depending on a number of determinants including unit’s volume, total nucleated cells (TNC) and total CD34^+^ cells, the latter two used as surrogate markers of the unit’s hemopoietic stem cell content. More recently, multiple studies have conclusively shown that the clinical outcome of CB transplant is positively associated with the hemopoietic stem cells administered dose. This evidence led to the identification of a progressively increasing threshold of the minimum TNC and CD34^+^ cell count required before starting expensive procedures for banking and long term cryopreservation, which currently can determine disposal rates of the collected CB units as high as 90% (Rafii et al., 2021). This high disposal rate can not only discourage donation but also significantly impact the economic sustainability of public CB banks. Moreover, the progressive implementation of successful HLA haploidentical transplant procedures in the last few years has been accompanied by a parallel decline in the number of CB transplants (Mayani et al., 2020), which further decreases the cost recovery of most CB banks. To overcome these difficulties, a number of projects have been developed within the CB banking community with the aim of improving the financial stability of public CB banks through the identification of improved banking strategies (Magalon et al., 2015; Hare et al., 2021; Wynn and Madrigal, 2021) and novel therapeutic CB uses (Querol et al., 2021; Scaradavou, 2021).

An international study aimed at identifying strategies for sustaining the economic future of public CB banks which used data from 28,473 CB units from four CB banks in France, Germany and the United States investigated four banking strategies based on CB unit “utilization rate” (the ratio of transplanted to banked units) and “utilization score” (as determined with a formula using TNC and CD34^+^ cell counts as its inputs) (Magalon et al., 2015). The authors determined the number of distributed transplants and the CB banks’ net cash flow using recruitment and processing costs reported in 2013 by the NMDP and the Swiss registries, under four scenarios of units with progressively increasing TNC and CD34^+^ cell counts: A: no use of the utilization score, mean TNC = 7.8 × 10^8^ and CD34^+^ cells = 2.5 × 10^6^, and 33% unit banking rate (9,396 banked/28,473 recruited); B: unit banking rate equal to 20% (5,695/28,473), mean TNC = 12.4 × 10^8^ and CD34^+^ cells = 4.3 × 10^6^; C: unit banking rate equal to 6% (1,708/28,473), mean TNC = 17.9 × 10^8^ and CD34^+^ cells = 6.7 × 10^6^; and D: unit banking rate equal to 2% (569/28,473), mean TNC = 24.8 × 10^8^ and CD34^+^ cells = 11.1 × 10^6^. Scenarios A and B, with the largest proportions of banked units, provided similar numbers of transplants (284 and 269 respectively), but unsustainable economic deficits of −5,886,986 and −3,046,101 USD respectively. Scenario D, with the lowest proportion of banked units, was associated with lower deficit (−2,189,089 USD), but this was associated with a significant reduction of the bank’s therapeutic value, i.e. the number of units distributed for transplant (*n* = 143). The authors considered scenario C as the most economically sustainable and therapeutically appropriate as it provided 219 transplants and a significantly lower deficit of −976,160 USD and concluded that “a pre-freezing level of 18 × 10^8^ TNC would be a cost-effective strategy to deliver therapeutic value to patients with a minimum financial deficit for the bank” (Magalon et al., 2015).

A “utilization-based” unit selection procedure using the formula originally developed by Magalon et al. (Rafii et al., 2021) was reported in 2021 by Wynn and Madrigal (Wynn and Madrigal, 2021), who produced a multi-parameter linear regression model integrating “eight predictive factors from pre-process flow cytometry and hematology data to generate a post-process predicted utilization score for each (CB unit) analyzed” (Wynn and Madrigal, 2021). The eight predictive factors included total granulocytes, lymphocytes, monocytes, nucleated red blood cells, CD34^+^ cells, volume, red blood cell count per microliter and age (hours) at reception. These authors identified five scenarios (A+, A, B, C and D, with progressively decreasing TNC and CD34^+^ cell counts) and determined that CB units graded A+ (*n* = 383/8296, with mean TNC = 28.8 × 10^8^ and CD34^+^ cells = 15.7 × 10^6^) accounted for 5% of total expenditures while providing 29% of all shipped grafts (65/227), as opposed to units graded D (*n* = 3168/8296, with mean TNC = 11.4 × 10^8^ and CD34^+^ cells = 5.2 × 10^6^), which accounted for 37% of processing cost despite being the source of only 11% of shipped transplants (24/227).

Careful evaluation by health professionals and patients’ advocates of the above methodologies in different banks and geographical jurisdictions can contribute to balancing the therapeutic value and the economic sustainability of public CB banking. Although the above evaluations can be improved by the optimization of CB collection, processing and cryopreservation, as shown in a recent report describing a novel collection procedure involving both *in utero* and ex utero collection of a single CB unit (Hare et al., 2021), the worldwide evidence shows that a large proportion of generously donated CB units cannot be *sustainably* used for their primary therapeutic application in hemopoietic stem cell transplants and are routinely wasted.

The aim of this article is to describe methodological, organizational and regulatory approaches developed in Italy and Spain to promote the development and clinical use of platelets, plasma and red blood cells from CB units not suitable for hemopoietic transplantation, a novel strategy collectively called “Multicomponent Cord Blood Banking”.

A detailed discussion of the *in vitro* uses and *in vivo* therapeutic applications of these novel CB components is outside the scope of this article. The interested reader can find the encouraging, preliminary evidences so far reported in several reviews (Petrini, 2012; Querol and Samarkanova, 2019; Rebulla et al., 2019; Samarkanova et al., 2020a) and laboratory studies (Parazzi et al., 2010; Cox et al., 2015; Parazzi et al., 2015; Ferri et al., 2016; Longo et al., 2016; Rebulla et al., 2016; Shirzad et al., 2017; Stokhuijzen et al., 2017; Christou et al., 2018; Cox et al., 2018; Valentini et al., 2019; Samarkanova et al., 2020b; Rallapalli et al., 2021). A selection of published investigations on the clinical use of CB platelets, plasma and red blood cells in wound healing, ophthalmology, neonatal and pediatric transfusion is reported in Table 1.

**TABLE 1:** A selection of published reports on the clinical use of CB platelets, plasma and red blood cells in wound healing, ophthalmology, neonatal and pediatric transfusion.

CB component	Product	Therapeutic field of use	Conditions	Ref
Platelets	Platelet Gel, Platelet Rich Plasma	Wound healing	Diabetic foot ulcers, pressure ulcers, epidermolysis bullosa, oral mucositis, fistulae, surgical wound dehiscence	Rosso et al. (2014); Piccin et al. (2017a); Piccin et al. (2017b); Volpe et al. (2017); Gelmetti et al. (2018); Sindici et al. (2018); Bisceglia et al. (2020); Volpe et al. (2020); Torkamaniha et al. (2021)
Platelets, Plasma	Eye Drops	Ophthalmology	Neurotrophic keratopathy, ocular graft versus host disease, ocular burns, dry eye syndrome, glaucoma	Erdem et al. (2014); Versura et al. (2014); Yoon (2014); Sharma et al. (2016); Giannaccare et al. (2017); Buzzi et al. (2018); Campos et al. (2018); Samarkanova et al. (2020c); Giannaccare et al. (2020); Samarkanova et al. (2021)
Red Blood Cells	Leukoreduced Red Blood Cells	Premature newborn’s and pediatric transfusion	Anemia	Bhattacharya et al. (2001); Bhattacharya (2006); Khodabux et al. (2008); Khodabux and Brand (2009); Khodabux et al. (2011); Hassall et al. (2012); Bianchi et al. (2015); Hassall et al. (2015); Bianchi et al. (2018); Neema (2018); Sarin et al. (2018); Teofili et al. (2018); Bianchi et al. (2020); González et al. (2020); Lopriore et al. (2020); Teofili et al. (2020)

### Italy

In Italy, CB banks (CBBs) are public hospital-based health facilities that collect, store, and distribute CB hematopoietic stem cells (HSC) on behalf of the National Health Service. CB banking, which was started in 1993, was formally organized into the Italian Cord Blood Network (ITCBN) in 2009, when a decree of the Ministry of Health assigned to the National Blood Center (Centro Nazionale Sangue, CNS) the coordination of the CB banking network in cooperation with the National Transplant Center (Centro Nazionale Trapianti, CNT) (Centro Nazionale Sangue, 2009a). The functions of the ITCBN are:

- To promote the allogeneic unrelated voluntary CB donation and meet the national and international CB demand for hematopoietic transplantation.
- To manage allogeneic related and autologous CB collection, storage and distribution for appropriate and evidence-based clinical indications.
- To promote the implementation of common standards and operative procedures. The ITCBN estimated an optimal national inventory of 60,000 units, based on scientific evidences that one unit per 1,000 inhabitants ensures that more than 90% of patients from the same ethnic group (Caucasians) can find a match to undergo an allogeneic transplant.

Eighteen ITCBN facilities are currently operating in 13 Italian regions, in compliance with national regulations (Centro Nazionale Sangue, 2005; Centro Nazionale Sangue, 2007; Gazzettaufficiale, 2010), guidelines and international standards (WMDA, NetCord-FACT). The ITCBN receives CB units from 270 delivery rooms and applies standard collection and banking requirements for volume, TNC and CD34^+^ cells. In July 2011, the ITCBN set a threshold of 12×10^8^ TNC pre-processing for allogeneic unrelated units, which was increased in 2016 to 16 × 10^8^ TNC pre-processing and 12 × 10^8^ TNC with 2 × 10^6^ CD34^+^ cells for banking. Up to December 2020, the total ITCBN inventory included 41,831 allogeneic unrelated units, 1,581 of which have been distributed to national and international transplant centers.

The CBBs belonging to the ITCBN are an integral part of the Regional Health Services, which are responsible for the organization and administration of publicly financed health care, including the cost of public CB banking. The reimbursement fee for one allogeneic unrelated CB unit amounts to 17,000 € (Centro Nazionale Sangue, 2015a). An ITCBN cost analysis showed that the main costs are related to cold chain maintenance, mostly determined by the costs of running cryogenic areas (Pupella et al., 2018), suggesting that scale economies can be obtained through centralisation of banking activities, as was done in several countries. The inventory target has not yet been achieved as well as the centralisation of banking activities. Furthermore, the significant drop of CB clinical use in recent years negatively affects the ITCBN sustainability. In this scenario, the CNS has promoted research projects for the development and regulation of new CB components.

Italian law no. 219 “New discipline of transfusion medicine activities and national production of blood derivatives” of 21 October 2005 includes CB donation and collection within blood transfusion activities (Centro Nazionale Sangue, 2005). CB banks release CB components based on a national cost reimbursement fee (Table 2). CB donation is voluntary, anonymous and not-remunerated, in agreement with the fundamental principle laid down by the blood transfusion law. Further technical decrees of the Ministry of Health have defined rules and requirements concerning CB donor selection and collection. In 2009, a Decree of the Ministry of Health established specific criteria for allogeneic unrelated HSC transplantation, allogeneic related HSC transplantation, and autologous/familiar therapeutic use according to scientific evidence based appropriateness, which is evaluated by a National Multidisciplinary Committee (Centro Nazionale Sangue, 2009b). In 2009 and in 2011, two official agreements between the National Government and the Regions defined minimum quality and safety standard requirements for CB activities and the national guidelines for CB bank accreditation (Centro Nazionale Sangue, 2017a; Centro Nazionale Sangue, 2017b).

**TABLE 2:** Reimbursement fees for the transfer of one unit of CB component between public health services applied at national level in Italy (Centro Nazionale Sangue, 2021).

Product (unit)	Fee (€)
Umbilical cord blood stem cells (*national fee established by the Italian Bone Marrow Donor Registry*)	17,000
Autologous/Allogeneic umbilical cord blood stem cells for approved family use	2,800
*Fee covers collection, manipulation, characterisation and biological validation, freezing, storage for 1 year, distribution excluding transport to the Transplant Center*	
Allogeneic cord blood-derived platelet concentrate for non-transfusional use	164
Platelet gel activation with Ca++	21
Platelet lysate used as eye drops (*amount sufficient for 1 month patient’s treatment*)	202

In 2014, considering the large availability of allogeneic CB units not suitable for transplant due to low HSC content, the ITCBN, under the coordination of the CNS, launched a national programme to standardise the preparation of a new CB component, allogeneic cord blood platelet concentrate (CBCP) suitable for the preparation of CB platelet gel (CBPG) (Rebulla et al., 2016). This programme yielded valuable scientific and operational information including CBPG cost calculation supporting the successive development of clinical trials. The first one was a prospective multicenter randomized clinical trial that treated patients affected by diabetic foot ulcers using the topical application of standardized CBPG gel in comparison to standard therapy (NCT02389010). Although this study was terminated due to financial limitations and low enrolment, it opened the door to the development of “CB multicomponent production” in some Italian CB banks. Several biological and clinical studies, still in progress, were able to provide preliminary scientific evidences of safety and efficacy of CB components as platelet gel, eye drops and red blood cells for neonatal transfusion.

In parallel, thanks to the collected scientific evidences, CB platelet concentrate, platelet gel and eye drops have been included in the transfusion technical norm, specifically the Decree of the Ministry of Health of 2 November 2015, integrated by the Decree of 1 August 2019 (Centro Nazionale Sangue, 2015b; Centro Nazionale Sangue, 2019). The latter allows the use of CB components for non-transfusional use not only for in-hospital applications but also for industrial manufacturing of medical devices and diagnostic kits. Furthermore, a dedicated official agreement has been established to harmonize the formal terms of a contract between blood establishment including CB banks and industrial manufacturers (Centro Nazionale Sangue, 2017c; Centro Nazionale Sangue, 2021). The implementation of specific reimbursement fees established at national level to cover the preparation costs sustained by the CB banks is in an advanced stage of development.

In the current worldwide public CB banking scenario, Italy is promoting a new hub-and-spoke ITCBN model. While a centralization of CB storage for transplant purposes in a small number of cryogenic facilities will improve the economic sustainability, maintenance of a large number of collection facilities will help increasing the HLA diversity of the national inventory. In a larger number of CB banks, preferably located in the transfusion services, a systematic multicomponent production could be implemented from a proportion of CB units unsuitable for HSC transplant. A regular production of the novel CB components might open the door to a regulated partnership with industrial stakeholders interested in manufacturing clinical grade biological drugs (e.g. eye drops).

### Spain

In Spain six public CB banks are in operation. These banks follow a national guide (Plan Nacional de Sangre de Cordón Umbilical–PNSCU) (ONT, 2021) aimed at harmonizing methods for collection, banking and release under the umbrella of the Spanish Bone Marrow Donor Registry (REDMO). Recently, a second version of the PNSCU has been approved for the period 2020-2025 with the following objectives:

- To meet the national CB inventory target of 60,000 CB units with at least 8 × 10^8^ TNC, including a stock renewal of units not meeting this TNC count threshold.
- To implement common quality criteria of the Spanish CB inventory.
- To develop research programs on new therapeutic uses of CB.
- To communicate to the society the benefit of public CB banking with regard to both the consolidate uses or the new potential applications.
- To design a common training and dissemination program on good clinical and manufacturing practices for health care professionals and regulatory bodies.

Following this guide, the Spanish CB network agreed that new units listed by REDMO should have a minimum post-processing count of 9 × 10^8^TNC, 2 × 10^6^ CD34^+^ cells, with a cell viability above 85%. This recommendation implies that units accepted for processing for transplantation should contain at least 15 × 10^8^ TNC and 4 × 10^6^ CD34^+^ cells. This very high threshold means that less than 20% of collected CB units will be further processed for transplantation and a substantial amount of collected unit will be wasted.

On December 31st, 2020, the Spanish CB inventory included 64,638 units, 77% of them meeting the agreed quality thresholds. Therefore, there is room for improving the inventory and CB banks are continuing collecting units. To increase this number to the desired target, the 6 CB programs have collected a median of 7142 (2365-13081) units per year during 2016–2020 (note that the advent of the COVID-19 pandemic and the associated restrictions have substantially reduced collection activities during 2020). From all collected units, only 13% (Petrini, 2012; Cox et al., 2015; Cox et al., 2018; Querol and Samarkanova, 2019; Rebulla et al., 2019; Samarkanova et al., 2020a; Samarkanova et al., 2020b) have finally been processed for transplantation.

The first CB unit available through REDMO was registered in april 1997 and the first shipment occurred on 5 May 1997, a unit sent to United States. Since then a total of 74,490 units have been transferred to REDMO and 86.7% of them are still available for clinical transplantation. During this time a total of 3,515 units were shipped for transplantation and 8.5% were deleted from the inventory due to various reasons (the most frequent (75%) after quality review during stability or reservation studies). The utilization rate in Spain is therefore 4.7% of the total registered inventory.

To estimate the cost of the national inventory, the data provided by Arrojo et al. (Arrojo et al., 2012), who estimated a production cost of 720,41 € per unit transferred to the REDMO, was used, suggesting that almost 53,600,000 € were invested in Spain to create the current CB inventory. Considering the utilization rate mentioned above, Spanish CB banks have spent 15,249 € per each CB unit provided for transplantation, making the program reasonably efficient.

Following the ratio of use decreased by 20% in the last 5 years and the re-orientation of PNSCU requiring researching in new applications, Barcelona CBB and the program Concordia BST, including the banking activity of six donation programmes in six autonomous regions of Spain plus Andorra, started the development of new therapeutic products from units not suitable for transplantation. In this regard a processing method to generate a new product based on CB platelets and plasma was validated (Samarkanova et al., 2020a). This new approach requires a regulatory overview on how those products can be used in Spain.

Blood donation and transfusion are subject to extensive regulation based on documents with different rank and scope at European and national level. In Spain, Royal Decree 1088/2005 (BOE, 2005) establishes the requirements, techniques and minimum standards for blood donation and blood establishments and sets the rules for the administration of allogeneic and autologous blood components. It gathers all Spanish regulations on blood donation and technical requirements, and incorporates the requirements included in the European regulations (Data Europa, 2002; Data Europa, 2004a). While this Royal Decree defines a blood component as any of the components of blood, including red blood cells, leukocytes, platelets, and plasma used for therapeutic purposes, progenitor cells, the industrial treatment of blood and its derivatives and the medicinal products resulting from it are excluded from the scope of the Royal Decree and are referred to their specific regulations. Some controversy has arisen over whether certain products derived from blood which are not used for transfusion are blood components or medicinal products, thus subject to the specific European and Spanish pharmaceutical regulations (Data Europa, 2001; Data Europa, 2004b; BOE, 2006; BOE, 2007).

In May 2013, the Spanish Medicines Agency (AEMPS) published a resolution establishing that autologous plasma and its fractions, components or derivatives are “medicinal products” but they fall under a separate category as they are used “to meet special needs” (AEMPS, 2021a). This resolution is based on the definition of medicinal product as “any substance or combination of substances presented for treating or preventing disease in human beings or which may be administered to human beings with a view to making a medical diagnosis or to restoring, correcting or modifying physiological functions in human beings” (BOE, 2006). As these regulations apply only to industrially produced medicinal products for human use, the AEMPS recognizes that medicinal products using autologous plasma should not be considered as industrially produced, and consequently should not be subject to the pharmaceutical regulations. Finally, and as defined in article five of Directive 2001/83/CE “*A Member State may, in accordance with legislation in force and to fulfil special needs, exclude from the provisions of this Directive medicinal products supplied in response to a bona fide unsolicited order, formulated in accordance with the specifications of an authorized health care professional and for use by his individual patients on his direct personal responsibility*” (Data Europa, 2001), the AEMPS classifies those autologous products as “medicinal products“ for human use “to meet special needs”.

While this resolution clarified the regulatory framework for autologous plasma and its derivatives, this is not the case for allogeneic products. In a questions and answers document, the AEMPS states that *non-substitutive therapeutic use of any other product based on non-autologous plasma would receive the treatment of biological medicine capable of being industrially produced and its authorization would be regulated by Royal Decree 1345/2007, of October 11, which regulates the procedure of authorization, registration and dispensing conditions of industrially manufactured medicines for human use,* so subject to the pharmaceutical regulations (AEMPS, 2021b).

Two medicinal products, a platelet gel from CB (CBPG) and an eye drop formula from cord blood platelet lysate (CBED) have been approved by AEMPS to be used in clinical trials. CBED was also employed in a programme of compassionate use in 33 patients (46 eyes) unresponsive to conventional treatments who required urgent intervention. Promptly available CBED resulted in a well-tolerated allogeneic treatment that showed evidence of efficacy (Samarkanova et al., 2020c). The reimbursement fees applied by the BST for the release of CB components are shown in Table 3.

**TABLE 3:** Reimbursement fees for the transfer of one unit of CB component applied by Banc de Sang i Teixits (www.bancsang.net).

Product (unit)	Fee (€)
Umbilical cord blood stem cells (*national fee established by the Spanish Bone Marrow Donor Registry*)	23,000
Autologous/Allogeneic umbilical cord blood stem cells for approved family use	2,471
*Fee covers collection, manipulation, characterisation and biological validation, freezing, storage for 1 year, distribution excluding transport to the Transplant Center* *^a^*	
Allogeneic cord blood-derived platelet concentrate for non-transfusional use including platelet gel activation with Ca++^a^	159.60
Platelet lysate used as eye drops (*amount sufficient for 19 days*)*^a^*	212.79

a In Spain there is no national fee for these products.

The different regulatory status of the same CB components in Italy (as blood components) and Spain (as medicinal products) indicates the need of regulatory harmonization. The current EU Directive on Blood (Data Europa, 2002) is under review by the European Commission and its evaluation report has been recently published (European Commission, 2019). Both platelet based products and serum eye drops are among the products identified with different classifications, from blood components in some countries to medicinal products in other countries or even though not regulated at all. The different national interpretation of industrial preparation or manufacturing is also highlighted as a cause of divergent classifications, which can explain why in Spain autologous based products have a different regulatory status as compared with non-autologous products.

## Conclusion

Multicomponent CB Banking using platelets, plasma and red blood cells from a proportion of currently discarded CB units could contribute to increasing the therapeutic value and reducing the financial deficit of public CB banking programs (Querol et al., 2021; Scaradavou, 2021). International regulatory harmonization and multicenter, large laboratory and clinical studies with sufficient statistical power are necessary to support the preliminary evidences reported in the literature (Bhattacharya et al., 2001; Bhattacharya, 2006; Khodabux et al., 2008; Khodabux and Brand, 2009; Khodabux et al., 2011; Hassall et al., 2012; Erdem et al., 2014; Rosso et al., 2014; Versura et al., 2014; Yoon, 2014; Bianchi et al., 2015; Hassall et al., 2015; Sharma et al., 2016; Piccin et al., 2017a; Piccin et al., 2017b; Giannaccare et al., 2017; Volpe et al., 2017; Bianchi et al., 2018; Buzzi et al., 2018; Campos et al., 2018; Gelmetti et al., 2018; Neema, 2018; Sarin et al., 2018; Sindici et al., 2018; Teofili et al., 2018; Bianchi et al., 2020; Bisceglia et al., 2020; Samarkanova et al., 2020c; Giannaccare et al., 2020; González et al., 2020; Lopriore et al., 2020; Teofili et al., 2020; Volpe et al., 2020; Samarkanova et al., 2021; Torkamaniha et al., 2021) and achieve these important goals.

## Data Availability

The original contributions presented in the study are included in the article/Supplementary material, further inquiries can be directed to the corresponding author.
